# Bacteremia Detection in Second or Subsequent Blood Cultures Among Hospitalized Patients in a Tertiary Care Hospital

**DOI:** 10.1001/jamanetworkopen.2022.8065

**Published:** 2022-04-20

**Authors:** Marie-Céline Zanella, Mikaël de Lorenzi-Tognon, Adrien Fischer, Nathalie Vernaz, Jacques Schrenzel

**Affiliations:** 1 Division of Infectious Diseases, Department of Medicine, Geneva University Hospitals, Geneva, Switzerland; 2 Bacteriology Laboratory, Department of Diagnostics, Geneva University Hospitals, Geneva, Switzerland; 3 Faculty of Medicine, University of Geneva, Geneva, Switzerland; 4 Medical Directorate, Geneva University Hospitals, Geneva, Switzerland; 5 Genomic Research Laboratory, Faculty of Medicine, University of Geneva, Geneva, Switzerland

## Abstract

This cohort study assesses the likelihood of detecting microbiological positivity or bacteremia in second or subsequent blood cultures among hospitalized patients while the first culture is still incubating after 24 hours.

## Introduction

Few data exist on the yield and utility of additional blood culture (BC) sets during extravascular infections. The low yield and false-positivity rate owing to contaminants during sample obtainment or handling^1^ support optimizing patient management and resource use. We assessed the clinical relevance of collecting an additional BC set while the first set is still incubating with results pending.

## Methods

This retrospective cohort study analyzed all BCs performed at a tertiary care hospital from January 1, 2019, through January 1, 2020, among adult patients with at least 2 BC sets collected during hospitalization, with the first collected in the emergency department within 24 hours after admission. Hematopoietic stem cell transplant recipients were excluded. The ethics committee of Geneva approved this study and waived informed consent owing to the retrospective design. We followed the STROBE reporting guideline.

We defined the first BC set as the first 2 bottles collected. Additional bottles were collected at least 24 hours later. Microbiological time to positivity (mTTP) was the time from start to end of incubation for a sample with a positive BC result (eFigure in the Supplement). The primary outcome was the likelihood of a positive BC result or detection of bacteremia with additional sets while the first was incubating after 24 hours, assessed using conditional probabilities for independent events (eMethods in the Supplement). Logistic regression and the Wald test assessed factors associated with bacteremia (secondary outcome). Kruskal-Wallis and χ^2^ tests compared continuous and categorical variables, respectively. Significance was set at 2-sided α = .05. Analyses were performed with R, version 4.1.0.

## Results

Among 23 088 BC bottles (2863 unique patients, 3214 care episodes) (Figure), the positivity rate was 8.34% (95% CI, 7.90%-8.70%), with 0.29% (95% CI, 0.28%-0.30%) of positive results owed to contaminants (Table). mTTP was 24 hours or less in 76.8% (95% CI, 74.8%-78.6%) of positive BC results, with mostly gram-negative bacilli and anaerobic microorganisms (Figure). Of 446 BC sets with mTTP longer than 24 hours, 317 (71.1%) were first sets (contaminants, 14.5%; 95% CI, 11.4%-17.9%) and 129 (28.9%) additional sets. In the latter, by care episode, the most common microorganisms were *Staphylococcus aureus* (34.8%; 95% CI, 22.7%-49.2%) and *Staphylococcus epidermidis* (8.7%; 95% CI, 3.4%-20.3%) and diagnoses were endovascular (58.7%; 95% CI, 44.3%-71.7%) and osteoarticular (19.6%; 95% CI, 10.7%-33.2%) infections. The probability of detecting bacteremia with additional BC sets was 4.1% (95% CI, 3.9%-4.4%) and 2.6% (95% CI, 2.4%-2.8%) when excluding contaminants and BC sets collected for endovascular infection. Female sex (odds ratio [OR], 0.34; 95% CI, 0.18-0.63; *P* < .001), hourly mTTP increment (OR, 0.98; 95% CI, 0.97-0.99; *P* = .005), sampling from a catheter (OR, 0.22; 95% CI, 0.10-0.47; *P* < .001), and growth of gram-positive microorganisms (OR, 0.04; 95% CI, 0.02-0.13; *P* < .001) were associated with lower odds for bacteremia.

**Figure.**
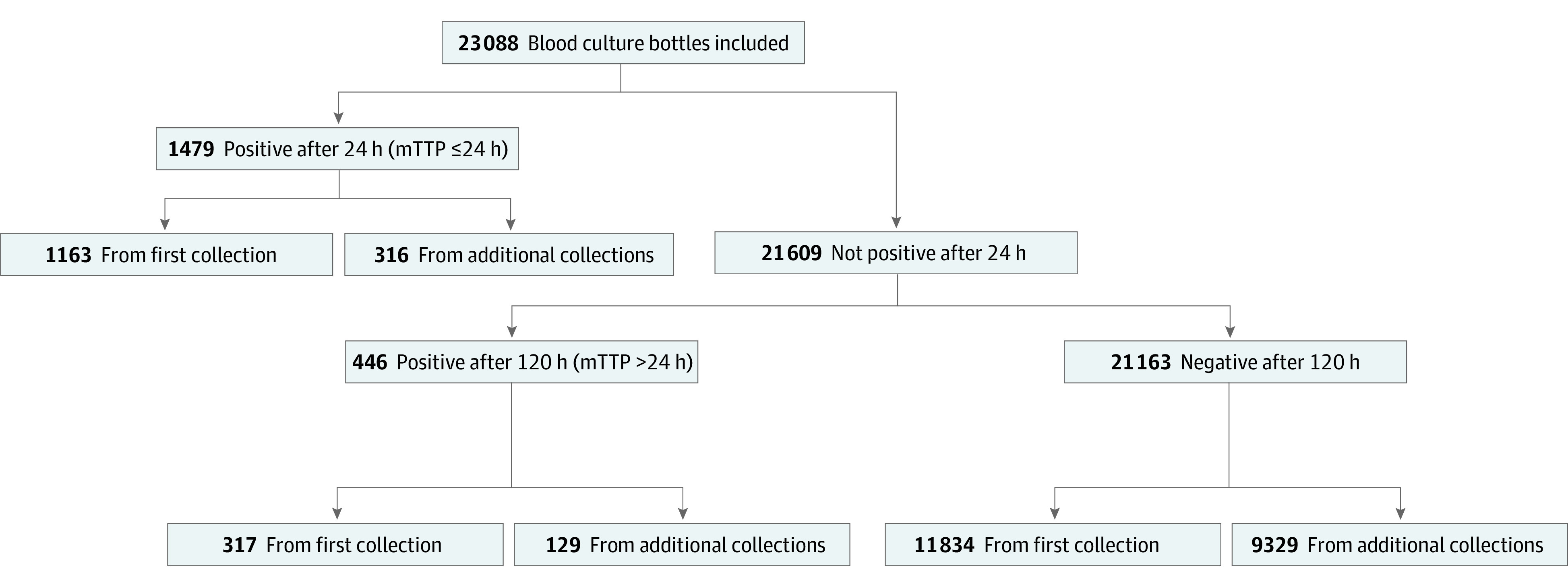
Flowchart of All Blood Culture Bottles Included and Stratified According to the Microbiological Time to Positivity (mTTP) and Culture Result

**Table.** Cohort Characteristics

Characteristic	BC bottles, No. (%)				
	Overall (N = 23 088)	Negative result (n = 21 163)	Positive result		
			mTTP≤24 h (n = 1479)	mTTP>24 h (n = 446)	*P* value^a^
Age, mean (SD), y	69.5 (18.3)	69.2 (18.5)	73.1 (15.3)	71.5 (15.7)	.07
Sex					
Female	10 446 (45.2)	9726 (46.0)	569 (38.5)	151 (33.9)	.09
Male	12 642 (54.8)	11 437 (54.0)	910 (61.5)	295 (66.1)	
Microscopic finding					
Gram-negative bacilli	1092 (4.7)	0	945 (63.9)	147 (33.0)	<.001
Gram-positive cocci	760 (3.3)	0	515 (34.8)	245 (54.9)	
Gram-positive bacilli	31 (0.1)	0	10 (0.7)	21 (4.7)	
Yeast	29 (0.1)	0	3 (0.2)	26 (5.8)	
Gram-negative cocci	12 (0.1)	0	6 (0.4)	6 (1.3)	
Gram-positive coccobacilli	1 (0.0)	0	0	1 (0.2)	
Solid-organ transplant recipient					
No	22 204 (96.2)	20 368 (96.2)	1413 (95.5)	423 (94.8)	.63
Yes	884 (3.8)	795 (3.8)	66 (4.5)	23 (5.2)	
Antimicrobial drugs received before BC sample collection, No.					
0	20 552 (89.0)	18 823 (88.9)	1318 (89.1)	411 (92.2)	.29
1	2005 (8.7)	1858 (8.8)	120 (8.1)	27 (6.1)	
2	493 (2.1)	445 (2.1)	40 (2.7)	8 (1.8)	
3	38 (0.2)	37 (0.2)	1 (0.1)	0 (0)	
Source of sample					
Peripheral venous puncture	21 116 (91.5)	19 406 (91.7)	1349 (91.2)	361 (80.9)	<.001
Via venous catheter	1611 (7.0)	1434 (6.8)	114 (7.7)	63 (14.1)	
Via arterial catheter	361 (1.6)	323 (1.5)	16 (1.1)	22 (4.9)	
Culture type					
Anaerobic bottle	11 283 (48.9)	10 373 (49.0)	752 (50.8)	158 (35.4)	<.001
Aerobic bottle	11 281 (48.9)	10 294 (48.6)	714 (48.3)	273 (61.2)	
Aerobic lytic bottle	524 (2.3)	496 (2.3)	13 (0.9)	15 (3.4)	
Preanalytical time, mean (SD), h^b^	5.15 (5.85)	5.18 (5.95)	4.44 (4.00)	6.24 (5.81)	<.001

Abbreviations: BC, blood culture; mTTP, microbiological time to positivity.

^a^ *P* value for the difference between the number of positive results at more than 24 hours vs 24 hours or less.

^b^ The preanalytical time corresponds to the time elapsed between the sample collection and the start of incubation and may be associated with laboratory operating hours.

## Discussion

The probability of detecting bacteremia with additional BC sets while the first was incubating after 24 hours was 4.1% (2.6% when excluding endovascular infections). The contamination rate was consistent with our institution’s rate for bloodstream infections. This quality-of-care indicator reflects adherence to the measures implemented in our institution for BC sample collection. Similar to other studies,^2,3,4^ most BCs (76.8%) had an mTTP of 24 hours or less. Preanalytical time was significantly higher for positive BC results with an mTTP greater than 24 hours.

A limitation was the retrospective design, which did not allow assessment of associations with outcomes. The review and extraction of data from medical records cannot exclude report and misclassification bias.

Campaigns such as Choosing Wisely and Smarter Medicine^5^ advocate avoiding unnecessary tests for patients, and medical resource preservation was highlighted by the COVID-19 pandemic, which challenged hospital and laboratory capacities.^6^ These findings support development of novel guidelines for BC sample collection to improve diagnostic resource use and patient management and to reduce costs and support rapid transportation and incubation of BCs to improve bacteremia diagnosis.

## Notes

Supplement.
eMethods.
**eFigure.** Illustration of the Study Set-Up and Different Parameter Definitions Used for the Analyses

## References

[1] Verboom DM, van de Groep K, Boel CHE, . The diagnostic yield of routine admission blood cultures in critically ill patients. Crit Care Med. 2021;49(1):60-69. doi:10.1097/CCM.0000000000004717 33165029

[2] Puerta-Alcalde P, Cardozo C, Suárez-Lledó M, . Current time-to-positivity of blood cultures in febrile neutropenia: a tool to be used in stewardship de-escalation strategies. Clin Microbiol Infect. 2019;25(4):447-453. doi:10.1016/j.cmi.2018.07.026 30096417

[3] Lambregts MMC, Bernards AT, van der Beek MT, Visser LG, de Boer MG. Time to positivity of blood cultures supports early re-evaluation of empiric broad-spectrum antimicrobial therapy. PLoS One. 2019;14(1):e0208819. doi:10.1371/journal.pone.0208819 30601829 PMC6314566

[4] Khatib R, Simeunovic G, Sharma M, . Blood culture series benefit may be limited to selected clinical conditions: time to reassess. Clin Microbiol Infect. 2015;21(4):332-336. doi:10.1016/j.cmi.2014.11.019 25658519

[5] Schweizerische Gesellschaft für Allgemeine Innere Medizin (Swiss Society for Internal Medicine). Smarter Medicine. 2021. Accessed February 24, 2021. https://www.smartermedicine.ch

[6] Haedo MF, Melendi SE, Lauko Mauri M, Ujeda C, Leis R. Usefulness of blood cultures in COVID-19 pneumonia. Medicina (B Aires). 2020;80(suppl 6):44-47. 33481732

