# Supplementary material for: Bacteremia Detection in Second or Subsequent Blood Cultures Among Hospitalized Patients in a Tertiary Care Hospital
Source: JAMA Netw Open. 2022 Apr 20;5(4):e228065. doi: 10.1001/jamanetworkopen.2022.8065 (PMC9021913; doi:10.1001/jamanetworkopen.2022.8065)
Supplement: Supplement. — eMethods. eFigure. Illustration of the Study Set-Up and Different Parameter Definitions Used for the Analyses [file jamanetwopen-e228065-s001.pdf]

Supplementary Online Content

Zanella MC, de Lorenzi-Tognon M, Fischer A, Vernaz N, Schrenzel J. Bacteremia detection in second or subsequent blood cultures among hospitalized patients in a tertiary care hospital. *JAMA Netw Open*. 2022;5(4):e228065. doi:10.1001/jamanetworkopen.2022.8065

eAppendix. eMethods
eFigure. Illustration of the Study Set-Up and Different Parameter Definitions Used for the Analyses

This supplementary material has been provided by the authors to give readers additional information about their work.

© 2022 Zanella MC et al. *JAMA Network Open*.

eAPPENDIX

eMethods

Microbiological Testing

Blood culture (BC) specimens were routinely inoculated into BACTEC® Aerobic Plus/F and Lytic Anaerobic/F bottles and incubated in the BACTEC® FX Blood Culture System (Becton-Dickinson, Sparks, MD, USA) for a maximum duration of 120 hours (5 days). For the Lytic MycoF bottles, the maximum incubation time was extended from 600 hours (25 days) to 720 hours (30 days) on September 1, 2019 (eFigure). Growing microorganisms were identified using standard laboratory procedures in our accredited laboratory using MALDI-TOF MS (MALDI Biotyper 2.0, Bruker, Germany). Antimicrobial susceptibilities were assessed by the disk diffusion method, in accordance with EUCAST guidelines and breakpoints (version 2019, https://www.eucast.org/fileadmin/src/media/PDFs/EUCAST_files/Breakpoint_tables/v_9.0_Breakpoint_Tables.pdf, accessed 17 Nov 2021).

Definitions

We defined the first BC set as the first two BC bottles collected in the ED (BC collected at t_0), whereas the additional BC bottles were collected at least 24 hours later. Each bottle was associated with date and time information for the following events: patient admission; BC collection; incubation start/end as reported by the automated blood culture system; and diffusion to the patient's electronic health record (EHR). The following values were derived: pre-analytical time (PAT) corresponding to the time elapsed between the sample collection and the start of incubation as it can be affected by laboratory operating hours, and microbiological time-to-positivity (mTTP) corresponding to the time elapsed between the start and the end of the incubation for a positive BC (eFigure). During the study period, our laboratory opening hours were 6:00am – 7:00pm. Outside this timeframe, BC were incubated

© 2022 Zanella MC et al. *JAMA Network Open*.

at room temperature in the general accessioning area until the next morning when they were loaded on the automated BC system.

Conditional Probabilities for Independent Events and Primary Outcome

Conditional probabilities for independent events were used to estimate the primary outcome.

$$P(B|A) = P(B) \times P(A)$$

Where P(A) is the probability that the first BC set is not positive after 24 hours of incubation; P(B) is the probability that an additional BC becomes positive within 5 days of incubation. P(B|A) is the probability that event B happens when event A has occurred.

The probability of detecting true bacteremia with additional BC sets was:

$$(1)\ P_1(A) = \frac{12\ 151}{13\ 314} = 0.912\ (CI95\%\ 0.908 - 0.917)$$

$$(2)\ P_1(B) = \frac{445}{9\ 774} = 0.045\ (CI95\%\ 0.041 - 0.049)$$

$$\mathbf{(3)\ P_1(B|A) = 0.045 \cdot\ 0.912 = 0.041\ (CI95\%\ 0.039 - 0.044)}$$

The probability of detecting true bacteremia when excluding contaminants and BC collected for endovascular infection was:

$$(1)\ P_2(A) = \frac{11\ 946}{13\ 004} = 0.918\ (CI95\%\ 0.913 - 0.923)$$

$$(2)\ P_2(B) = \frac{266}{8\ 957} = 0.029\ (CI95\%\ 0.026 - 0.033)$$

$$\mathbf{(3)\ P_2(B|A) = 0.029 \cdot\ 0.918 = 0.026\ (CI95\%\ 0.024 - 0.028)}$$

Data Extraction and Processing

Data were extracted from the EHR to create a specific study database and then processed to filter out non-relevant data. The final table included 43 variables for 23 088 rows, each row corresponding to one BC bottle collected during the study period, and thus describing one observation. The following variables were collected: source of BC collection; culture result;

© 2022 Zanella MC et al. *JAMA Network Open*.

patient ward at time of BC collection; transplantation status; current antimicrobial therapy; microorganisms identified in the positive BC bottle; patient age and gender.

EHR Review and Screening Process

We screened our final table for potential BC contaminants whenever culture reported one of the following microorganisms: *Corynebacterium* spp, *Cutibacterium acnes* and coagulase-negative staphylococci. Each care episode reporting at least one of these microorganisms prompted an EHR chart review. The contaminant was then confirmed and flagged whenever it was clearly documented in the EHR, either by the infectious diseases' consultant or the physician in charge of the patient.

Statistical Analysis

We used the Kruskal-Wallis one-way analysis of variance for numeric variables and the Chi-squared test for categorical variables; p-values are shown with a maximum of 3 decimals and the level for statistical significance was set at $P \leq .05$ (two-sided), if not specified otherwise. Due to the non-negligible probability to get a type I error caused by the multiple comparison in subgroup analyses for the performance assay, we used $P \leq .005$ as the cut-off for significance by applying the Bonferroni correction. McNemar's test was used to assess the statistical significance in sensitivity and specificity changes as depicted by Fagan's nomogram. All data and statistical analyses were performed using R software version 4.1.0 (2021-05-18) and the following extension packages: dplyr, table1, bdpv, DTComPair, caret, ggplot2, pROC, epiDisplay and MASS in their latest available version.

Multivariate Logistic Regression, Adjusted Odds Ratios and ROC Curve

A new variable named 'bacteremia' was inserted into the dataset and attributed the value of 0 in the absence of true bacteremia. Thus, a contaminant would be attributed the value of 0, despite being reported as a positive BC bottle. This was used as the dependent variable to perform multivariate logistic regression to assess multiple predictor variables. To increase

© 2022 Zanella MC et al. *JAMA Network Open*.

the accuracy of our model, we performed a cross-validation by training it using 80% of the data and kept the remaining 20% to assess its performance. The best fitting model (AIC=391.5) included: mTTP; source of sampling (i.e., intravenous/intra-arterial line or peripheral venous punctures); gender; transplantation status; direct examination under the microscope for microorganisms (e.g., morphology, Gram staining or identification as a yeast); and culture type (aerobic, anaerobic, Lytic MycoF). Adjusted odds ratios (aOR) were calculated with the corresponding 95% CI.

FIGURE

eFigure. Illustration of the Study Set-Up and Different Parameter Definitions Used for the Analyses.

BC: blood cultures; PAT: pre-analytical time; mTTP: microbiological time-to-positivity; EHR: electronic health record.

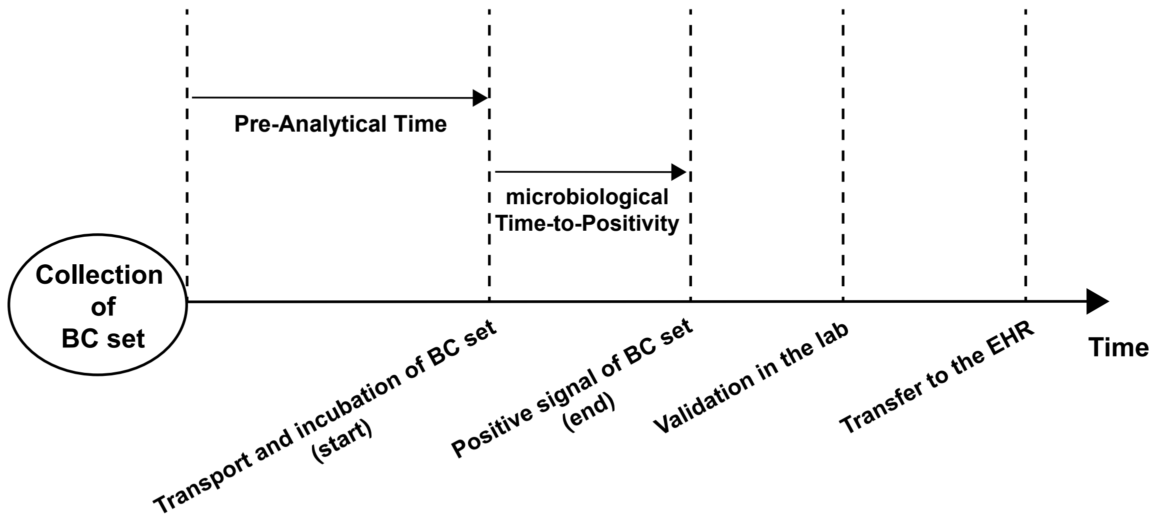

© 2022 Zanella MC et al. *JAMA Network Open*.
